# First person – Mingkai Zhu

**DOI:** 10.1242/dmm.052161

**Published:** 2024-10-30

## Abstract

First Person is a series of interviews with the first authors of a selection of papers published in Disease Models & Mechanisms, helping researchers promote themselves alongside their papers. Mingkai Zhu is first author on ‘ Sex hormone receptors, calcium-binding protein and Yap1 signaling regulate sex-dependent liver cell proliferation following partial hepatectomy’, published in DMM. Mingkai conducted the research described in this article while a PhD student in Dr Dong Liu’s lab at Southern University of Science and Technology, Shenzhen, China and Dr Zhiyuan Gong's lab at National University of Singapore, Singapore. Mingkai is now a research fellow in the lab of Dr Wolfram Goessling at Massachusetts General Hospital, Boston, MA, USA, investigating regenerative medicine.


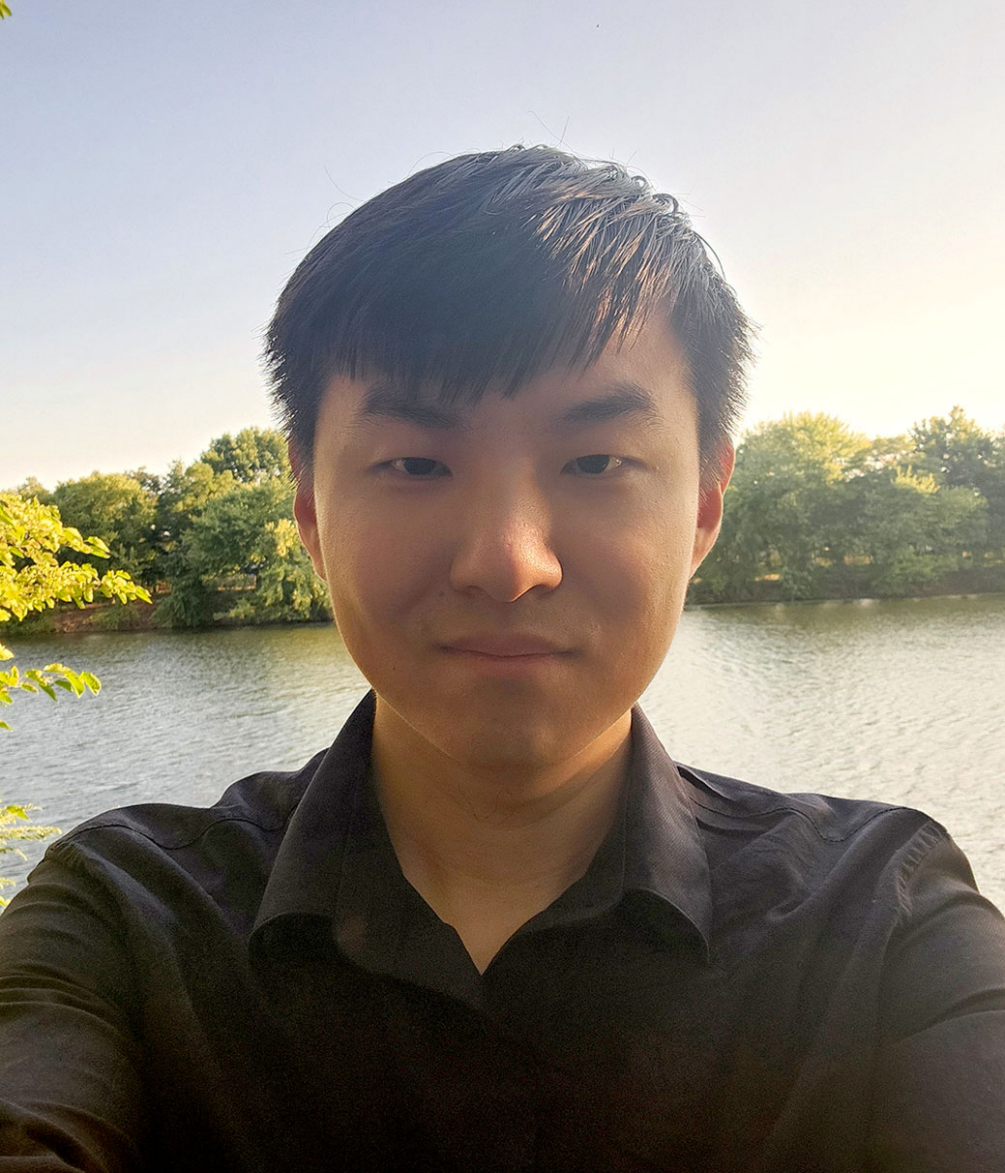


**Mingkai Zhu**

**Who or what inspired you to become a scientist?**

My interests in biology and medicine originated early as my parents were medical professionals and often shared their stories in the hospital with me. Back in high school, my grades in biology were top of the class, which raised my confidence and made me think I may have some talent in this subject. With encouragement from my parents, I majored in biology at university and later went overseas to pursue further degrees. The study of induced pluripotent stem cells by Shinya Yamanaka inspired my research interest in regenerative medicine when he was awarded the Nobel Prize, and later helped me to choose my research projects. During my PhD training, although sometimes the experiments could be frustrating, I still enjoyed my research because I felt that my work could be beneficial to human knowledge. So, I decided to stay in the academic field after my PhD.

[…] we identified the calcium-binding protein S100A1 as a potential regulator of sex-biased liver regeneration in zebrafish.

**What is the main question or challenge in disease biology you are addressing in this paper? How did you go about investigating your question or challenge?**

Sex has been reported to affect the recovery of patients from liver operations in the clinic. Previous studies have demonstrated the existence of sex disparity in the speed of liver regeneration in rodents following partial hepatectomy (PH). However, the mechanism behind this sex disparity has not been sufficiently explored. This study aims to better understand how sex affects liver regeneration following PH and the mechanism underlying this sex disparity using the zebrafish PH model. We first confirmed the sex disparity in PH-induced liver regeneration in zebrafish by comparing the regenerative process between males and females in terms of liver cell proliferation and liver mass recovery. We found that this sex disparity in liver regeneration was associated with sex-biased activation of Yap1 signaling following PH. Then, we examined how the activities of sex hormone receptors regulate the sex disparity in liver regeneration following PH by altering the activities of oestrogen and androgen receptors with chemicals. Meanwhile, we also looked for other factors that are potentially involved in this sex disparity by RNA sequencing. Based on the RNA-sequencing and real-time quantitative PCR results, we identified the calcium-binding protein S100A1 as a potential regulator of sex-biased liver regeneration in zebrafish. We hypothesized that S100A1 regulates the sex disparity in zebrafish liver regeneration by regulating Yap1 activity. To verify this hypothesis, we conducted morpholino-mediated knockdown of *s100a1* in larvae and observed the effects of *s100a1* knockdown on liver development and liver regeneration after chemical-induced injury. Furthermore, we generated a *s100a1*-knockout line with CRISPR/Cas9-mediated large-fragment deletion to confirm the roles played by *s100a1* in adult zebrafish liver regeneration following PH.

**How would you explain the main findings of your paper to non-scientific family and friends?**

A common treatment in the clinic for patients with liver cancer is to surgically remove the tumour-bearing part of the liver. As the human liver has the ability to regenerate its mass, patients’ liver function can recover from this type of surgery. However, compromised liver regeneration after liver resection can lead to liver failure, which is life threatening. It is known that sex can affect liver regeneration, but the mechanism behind this effect is largely unclear. In this study, we explored this topic with zebrafish – a freshwater fish widely used in regeneration studies. We found that male zebrafish start liver regeneration earlier than female zebrafish after they receive liver resection. This male-biased initiation of liver regeneration requires the participation of multiple biomolecules, including sex hormone receptors and other proteins named Yap1 and S100A1. Some of them, such as Yap1 and S100A1, work together to facilitate liver regeneration in male zebrafish after liver resection.

**What are the potential implications of these results for disease biology and the possible impact on patients?**

In the clinic, female patients usually have a better prognosis than male patients after receiving liver resection. This phenomenon, together with the sex-biased liver regeneration demonstrated by animal studies, suggests the need for a finely formulated therapeutic strategy based on sex disparity for patients who require liver resection. Our findings in this study provide more insights into the sex disparity in liver regeneration and the regulatory roles played by sex hormones and the S100A1-YAP1 signaling cascade in this process, which will help the development of novel treatments corresponding to patient sex. Aside from sex disparity, the identification of the promoting role played by S100A1 in liver regeneration provides a novel target for drug development.

**Figure:**
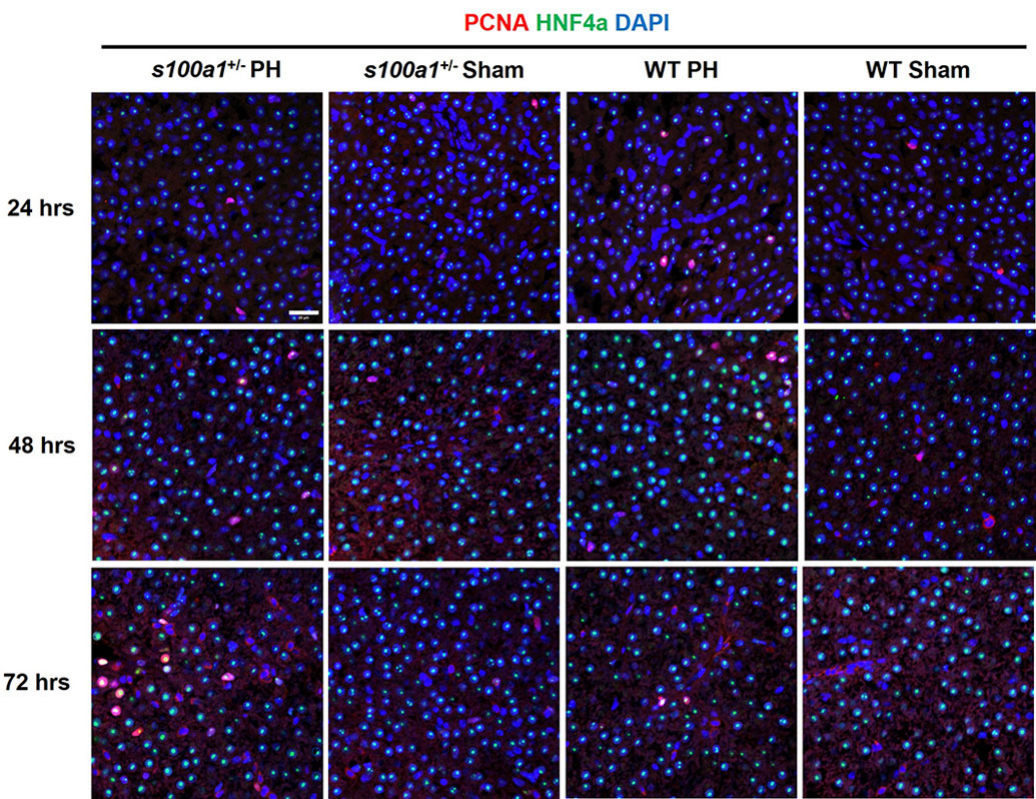
**S100A1 deficiency delays hepatocyte proliferation in male zebrafish livers after partial hepatectomy (PH).** Immunofluorescence staining of Pcna and Hnf4α in *s100a1*^+/−^ and wild-type (WT) male zebrafish livers at 24, 48 and 72 h after PH/sham surgery. The increase in the number of Pcna/Hnf4α double-positive proliferating hepatocytes at 24 h post-PH was inhibited in the *s100a1*^+/−^ fish. Scale bar: 20 µm.

**Why did you choose DMM for your paper?**

One of my PhD supervisors recommended DMM to me and said it has a good reputation and suits the topic of our manuscript. Our lab has also published multiple high-quality papers in DMM previously. In addition, this study used the zebrafish PH model, which is a relatively mature model but has only been applied in a limited number of studies. I think DMM will provide a good platform for more researchers to learn about this useful model.

**Given your current role, what challenges do you face and what changes could improve the professional lives of other scientists in this role?**

I just started my job as a postdoc 3 months ago. So, I am not yet experienced enough to give an opinion on the challenges postdocs face in general. However, I did meet a systematic problem when publishing my PhD research after graduation. When we were submitting the manuscript of this paper, one of my supervisors was close to retirement and would lose access to his funding soon, which made him unable to pay the processing fee for this paper. Fortunately, I have been working in two labs during my PhD, and another supervisor submitted the manuscript for me. I am sure that most PhD students do not have multiple PIs who can pay for their publication, and that it is not uncommon for them to publish their work after completing their degree. Allowing PIs to use funding to publish old results after their retirement could be a more flexible way to solve this issue.

**What's next for you?**

Currently, I am working at Massachusetts General Hospital as a research fellow. In my new lab, my research focus is still liver regeneration, and I am still working with zebrafish. However, my current project is about other aspects of liver regeneration, such as the involvement of liver progenitor cells and the conversion between parenchymal and non-parenchymal cells during regeneration.

**Tell us something interesting about yourself that wouldn't be on your CV**

I failed constantly in English classes during middle school, and my English became better only after high school. So, back then, I could not imagine someday being in a position that required me to read and write in English on a daily basis.
